# An individualized predictor of health and disease using paired reference and target samples

**DOI:** 10.1186/s12859-016-0889-9

**Published:** 2016-01-22

**Authors:** Tzu-Yu Liu, Thomas Burke, Lawrence P. Park, Christopher W. Woods, Aimee K. Zaas, Geoffrey S. Ginsburg, Alfred O. Hero

**Affiliations:** Electrical Engineering and Computer Science Department, University of California, Berkeley CA, USA; Center for Applied Genomics and Precision Medicine, Department of Medicine, Duke University, Durham NC, USA; Electrical Engineering and Computer Science Department, University of Michigan, Ann Arbor MI, USA; Center for Computational Biology and Bioinformatics, University of Michigan, Ann Arbor MI, USA

**Keywords:** Reference-aided prediction, Precision medicine, Automated diagnostics, Biomarker discovery, Sparse multi-block classifier algorithm

## Abstract

**Background:**

Consider the problem of designing a panel of complex biomarkers to predict a patient’s health or disease state when one can pair his or her current test sample, called a target sample, with the patient’s previously acquired healthy sample, called a reference sample. As contrasted to a population averaged reference this reference sample is individualized. Automated predictor algorithms that compare and contrast the paired samples to each other could result in a new generation of test panels that compare to a person’s healthy reference to enhance predictive accuracy. This paper develops such an individualized predictor and illustrates the added value of including the healthy reference for design of predictive gene expression panels.

**Results:**

The objective is to predict each subject’s state of infection, e.g., neither exposed nor infected, exposed but not infected, pre-acute phase of infection, acute phase of infection, post-acute phase of infection. Using gene microarray data collected in a large scale serially sampled respiratory virus challenge study we quantify the diagnostic advantage of pairing a person’s baseline reference with his or her target sample. The full study consists of 2886 microarray chips assaying 12,023 genes of 151 human volunteer subjects under 4 different inoculation regimes (HRV, RSV, H1N1, H3N2). We train (with cross-validation) reference-aided sparse multi-class classifier algorithms on this data to show that inclusion of a subject’s reference sample can improve prediction accuracy by as much as 14 %, for the H3N2 cohort, and by at least 6 %, for the H1N1 cohort. Remarkably, these gains in accuracy are achieved by using smaller panels of genes, e.g., 39 % fewer for H3N2 and 31 % fewer for H1N1. The biomarkers selected by the predictors fall into two categories: 1) contrasting genes that tend to differentially express between target and reference samples over the population; 2) reinforcement genes that remain constant over the two samples, which function as housekeeping normalization genes. Many of these genes are common to all 4 viruses and their roles in the predictor elucidate the function that they play in differentiating the different states of host immune response.

**Conclusions:**

If one uses a suitable mathematical prediction algorithm, inclusion of a healthy reference in biomarker diagnostic testing can potentially improve accuracy of disease prediction with fewer biomarkers.

**Electronic supplementary material:**

The online version of this article (doi:10.1186/s12859-016-0889-9) contains supplementary material, which is available to authorized users.

## Background

It is evident that that patient history can improve interpretability of diagnostic data such as a panel of assayed biomarkers. When this history includes a previously collected assay, the assay constitutes a reference baseline against which the current assay can be quantitatively compared. However, as the size and complexity of clinical biomarker panels increase, manual cross-assay comparisons become impractical. This motivates the development of automated algorithms that can combine a current target assay and a reference assay with improved prediction or classification performance. In this paper we consider the problem of using a panel of biomarkers to predict a patient’s health state when both the target sample and reference sample are available. Two questions are of interest. Can such a reference sample be used to more accurately assess the deviation of the target sample from a previously established patient baseline, potentially translating into improved predictions? Can such predictions be performed accurately with relatively fewer biomarkers, i.e., a smaller test panel, potentially translating into a less expensive test? In this paper we show that the answer to both of these questions is affirmative. Using a state-of-the-art multi-block sparse predictor algorithm, and a large-scale serially sampled data set collected in a human viral challenge study, we present an algorithm for reference-aided health prediction that attains higher predictive accuracy using a smaller panel of biomarkers.

The reader may not find it surprising that automated diagnostics may benefit from pairing a reference sample and a target sample. Indeed, it has been common clinical practice for a physician to manually compare a small number of a patient’s analytes to his or her previous test results. However, such manual comparison will become increasingly difficult as we enter the era of precision medicine where whole genome expression or next generation sequencing platforms may play an important clinical role [1–3]. In this era, automated algorithms will be needed not only for accurate prediction but also for selection of a suitably small subset of the thousands of probes generated by these platforms. Such algorithms impose sparsity on the predictor by utilizing only a small fraction of the available probes. The reduction of the number of probes (genes) is relevant to personalized medicine applications since it leads to a more economical (lower complexity) targeted biomarker assay. Previous work has developed such algorithms in the context of prediction of acute respiratory virus infection [4–6]. This paper goes one step further and shows that adding one healthy reference sample can result in improved prediction performance.

The paper is organized as follows. We first present the formulation of our optimization problem in the “Methods” section, including the loss function used as surrogates in reference-based classification, the proper regularization that selects variables relevant simultaneously to all classes and references, and followed by a discussion about the general algorithm we propose to solve the optimization. Then we present the performance of the reference-based classification applied to H3N2, H1N1, HRV, and RSV flu challenge data sets in the results section. Advantages of the methods and biological interpretation are presented in the discussion section. The conclusion section concludes this paper.

## Methods

The proposed reference-aided prediction method is based on a state-of-the-art supervised multi-block multi-class classifier algorithm with variable selection [7]. To illustrate the advantages of the proposed predictor, we will demonstrate superior prediction performance on data collected from large scale serially sampled respiratory virus challenge studies. Data from the challenge studies have previously been used by us and others to derive molecular signatures for acute respiratory infection (ARI) [4, 5, 8, 9]. This paper’s contribution is the introduction of a new individualized reference-aided predictor that is demonstrated on an extended set of data collected from additional challenge studies (see Table 1). More details on these challenge studies can be found in the aforemementioned references and in the Additional file 1. We describe the challenge studies first and then turn to the automated predictor afterwards.

**Table 1:** Composition of data collected in the respiratory virus challenge study. The study enrolled a total of 151 subjects challenged with 4 difference viruses over seven different challenge sub-studies and samples at multiple regularly spaced time points over a time period ranging from 3–5 days. The first column is the sub-study designation. Second column is the virus used in the challenge. Third and fourth columns are the year and location the sub-study was conducted. Fifth column is the DUHS IRB protocol number. Sixth column is the duration of the sub-study in hours. Last two columns are the number of subjects and the number of time points collected per subject, respectively

Challenge	Virus	Year	Location	IRB protocol	Duration (hrs)	# Subjects	# Time points
DEE1	RSV	2008	Retroscreen	Pro00002796	166	20	21
DEE2	H3N2	2009	Retroscreen	Pro00006750	166	17	21
DEE3	H1N1	2009	Retroscreen	Pro00018132	166	24	20
DEE4	H1N1	2010	Retroscreen	Pro00019238	166	19	21
DEE5	H3N2	2011	Retroscreen	Pro00029521	680	21	23
HRV UVA	HRV	2008	Univ. of Virginia	Pro00003477	120	20	15
HRV Duke	HRV	2010	Duke Univ.	Pro00022448	136	30	19

### Viral challenge study model

To demonstrate the advantages of reference-based prediction, we use data from a serially sampled challenge study. The challenge study consists of a total of 151 subjects (human volunteers) that, shortly after enrollment in the study, were inoculated with sham or live virus from one of 4 categories of pathogen (HRV, RSV, H3N2, H1N1). The overall study was conducted over a 4 year time period in 7 stages (see Table 1 for a summary). Research participants in these studies provided informed consent and all research activities were conducted in accordance with the Declaration of Helsinki and local policies and regulations. These studies were approved by the Duke University Health System (DUHS) Institutional Review Board (IRB). Where applicable, additional approval was obtained from a local governing IRB where the study activities occurred: Western Institutional Review Board (WIRB) and the University of Virginia IRB approved the studies that were conducted Retroscreen Virology, London, UK and UVA, respectively.

Each subject in the study was serially sampled for several days quantifying time courses of whole blood gene expression by Affymetrix Human U133A 2.0 GeneChips, self-reported clinical symptom scores over 8-10 symptoms (varied by study), and viral shedding from periodic nasopharyngeal titrations. The Affymetrix gene probes were log transformed and normalized using the RMA package with quantile normalization, median polish and a custom cdf mapping from oligoprobes to gene yielding 12,023 gene probes (see Additional file 1 for details on genechip normalization and symptom symptom score definitions).

Subjects were sampled at least once before the viral inoculum was administered and at least 14 times after inoculation. Each subject was designated as a symptomatic subject (Sx) or an asymptomatic subject (Asx) and as an infected subject (Inf) or uninfected subject (UnInf). The Asx/Sx designation was based on a modified Jackson score computed from the self-reported clinical symptoms [10, 11]. The Inf/UnInf designation was determined from viral shedding data: a subject was declared infected if the viral titers exceed a high threshold at any time point or if they exceed a lower threshold at any tow time points. Further details are provided in the Additional file 1 deposited to the GEO database (accession number GSE73072).

For the prediction analysis, we excluded 44 clinically ambiguous subjects due to inconsistencies between their declared symptomatic status and measured shedding status and 3 subjects that had no Affymetrix gene probes collected. These 44 clinically ambiguous subjects were at some time either acutely infected but asymptomatic or not infected but acutely symptomatic. Thus the results reported below are restricted to the 104 unambiguously healthy (Asx and uninfected) and unambiguously ill (Sx and infected) subjects. Of these 104 unambiguous subjects 41 were infected subjects and 63 were uninfected subjects, and they will be designated as such in the sequel.

For these 104 subjects five time-specific infection states were determined on the basis of symptom scores and the viral shedding measurements. State 1 is “baseline” before inoculation. The other states occur after inoculation. State 2 is “Asx and UnInf” and applies to all post-inoculation samples of the uninfected subjects. States 3, 4 and 5 occur in the infected subjects after inoculation. State 3 is “Sx and pre-acute Inf,” State 4 is “Sx and acute Inf,” and State 5 is “Sx and post-acute Inf.” For each subject a healthy reference genechip sample was taken from baseline (state 1) and paired with one of the post-inoculation genechip samples taken from the subject’s post-inoculation time course (states 2-5). The state predictors, described below, were trained and tested on subsets of these paired samples. The 5 state designations are illustrated in Fig. 2c for the H3N2 DEE2 cohort and in corresponding figures for the HRV, RSV and H1N1 cohorts availabel on GEO (GSE73072). The Additional file 1 also contains details about the data collection and the criteria used for designating the states from shedding and symptom score data.

**Fig. 1:**
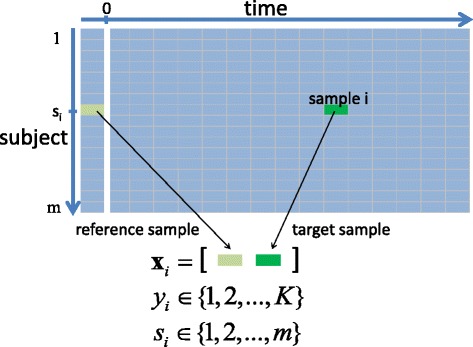
Sample data layout for standard and reference-aided prediction. Each cell in this matrix corresponds to a sample **x**_*i*_ of a subject *s*_*i*_ taken at some time during the viral challenge study. The corresponding infection state label *y*_*i*_ for each target sample **x**_*i*_ is shown in Fig. 2.C for the particular case of the H3N2 challenge study. The standard predictor tries to predict the state using only the target sample. The reference-aided predictor uses both the target sample and the reference sample taken prior to inoculation time, denoted by the white vertical line

**Fig. 2:**
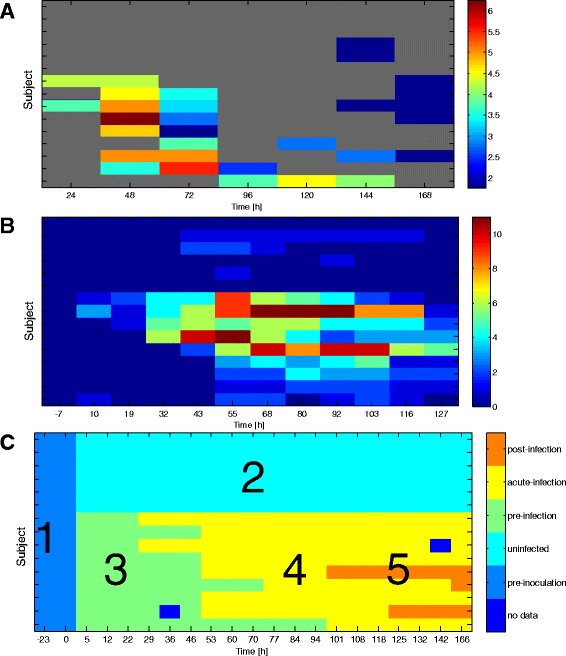
H3N2 DEE2 challenge study viral shedding map (**a**), symptom score map (**b**), infection state map (**a**). **a** shows viral titration measurements for each subject at each sample time. **b** shows the sum of the 10 self-reported symptom scores. We use the measurements in A and B to designate each subject as infected (Inf), noninfected (UnInf), symptomatic (Sx), or asymptomatic (Asx). The subject dedignations can be found in the Additional file 1. Subjects whose titer scores and symptom scores agree, i.e., those who are either infected and symptomatic or uninfected and asymptomatic, are used for training the predictors. We assign 5 state labels to these subjects, as shown in (**c**), that correspond to baseline reference (state 1), UnInf (state 2), pre-accute Inf (state 3), acute Inf (state 4), and post-acute Inf (state 5). The onset and offset time of detectable titration are used to set the boundaries between class 3 and 4 and class 4 and 5 respectively (see Additional file 1)

Mathematically, we denote the *i*-th microarray sample as the *p*=12,023 dimensional vector **x**_*i*_. The *i*-th sample is labeled as *y*_*i*_∈{1,2,…,*K*} corresponding to one of the *K*=4 possible infected/symptomatic states. The subject from whom the *i*-th sample was collected is denoted as *s*_*i*_∈{1,…,*m*}, where *m* is the total number of subjects. Figure 1 illustrates the time vs. subject matrix layout of the challenge study. The time instant labeled 0 (white vertical line) corresponds to the time of inoculation. The location of a hypothetical reference sample and target sample for a given subject *s*_*i*_ is shown in the figure. For illustration, Fig. 2 shows the titration and symptom data collected from subjects in the H3N2 DEE2 study. Similar figures for the other studies, summarized in Table 1, are available on GEO (GSE73072).

### Prediction algorithms

To establish and quantify the value of including a subject’s reference sample, we implement a state-of-the-art automated prediction algorithm that performs variable selection and accomodates a reference sample in addition to a target sample. The predictor for the state *y*_*i*_ is learned from the biomarker data **x**_*i*_ using a supervised sparse multi-block multi-class classification algorithm, described in detail below. The different classes classified by the algorithm correspond to the different infection states. Sparsity forces the algorithm to select a small number of biomarkers (genes) from the 12,023 possible biomarkers. The imposition of sparsity is required in order to minimize overfitting error since the number of samples available to train the classifier is much smaller than the total number of biomarkers [12, 13]. The multi-block structure is used to force the reference-aided classifier to use the same subset of biomarkers for the paired reference and target samples in the classifier function. More specifically, as discussed below, for the reference-aided predictor there are two blocks corresponding to, respectively, the gene probe values in the reference sample and the target sample. For the standard predictor there is only one block corresponding to the gene probe values of the target sample.

A classifier is a function that operates on a data point **x** (the input) and produces a decision $\hat {y}$ (the output) about the class, where $\hat {y}\in \{1, \ldots, K\}$. In machine learning the classifier function is optimized to achieve the best possible classification accuracy over a set of training samples $\{ {\mathbf x}_{i}, y_{i}\}_{i=1}^{n}$, which are typically a subset (the training set) of all the available data. The result of this optimization is often averaged over many different training subsets of the data, e.g., by random resampling or leave-one-out resampling, a process called classifier cross-validation [12]. Among the many different algorithms available for multi-class classification the support vector machine (SVM) is one of the most prevalent. There are two common strategies for multi-class classification that have been proposed: (1) solving the multi-class problem by a series of binary SVM classifiers [14, 15]; (2) formulating a single unified multi-class SVM [16–21]. In this paper we adopt the latter more direct approach to multi-class classification.

As described in [19], the unified *K*-class classifier is a scoring based algorithm that classifies the input by computing its score for each class and outputs the class label associated with the maximum score. Specifically, given *K* functions *f*_1_,…,*f*_*K*_ the unified *K*-class classifier outputs the decision $\hat {y}=\arg \max _{k} f_{k}(\mathbf x)$ These functions assign confidence scores to the input **x** and can be chosen as linear functions of the form

(1) $$ f_{k} (\textbf{x}) = {\textbf{w}_{k}}^{T} \textbf{x} + b_{k}, \hspace{1in} k=1, \ldots, K   $$

where $\mathbf {w}_{k}\in \mathbb R^{p}$ is a (column) vector of *p* weights $\{w_{\textit {ki}}\}_{i=1}^{p}$ and $b_{k}\in \mathbb R$ is a scalar offset. While other forms of the score functions are also common, e.g., kernelized linear, polynomial or sigmoidal functions, we will use the linear function (1) to design both the standard and reference-aided predictor.

Since there are many fewer samples (*n*) than variables (*p*) it is desirable to reduce the number of biomarkers used by the classifier in order to minimize overfitting errors [12]. This can be accomplished by constraining the weight vectors $\mathbf {\{w_{k}\}}_{\mathbf {k=1}}^{\mathbf {K}}$ to have common sparsity, i.e., the **w**_*k*_’s have many common entries equal to zero. Defining the *K*×*p* weight matrix **W**=[**w**_1_,…,**w**_*K*_]^*T*^ and *K*-element vector $\bf b= \left [b_{1}, \ldots, b_{K}\right ]^{T}$, this common sparsity constraint is expressed as **W** having many columns identically equal to zero. This is a form of structured sparsity [22], also called group sparsity, that is mathematically expressed as the “mixed *ℓ*_1_/*ℓ*_0_ norm” constraint on **W**: $\|\mathbf {W}\|_{1,0}=\sum _{j=1}^{p} \|\mathbf {w}_{(j)}\|_{0} \leq q$, where *q* is much less than *p*, **w**_(*j*)_ is the *j*-th column of **W** and ∥*u*∥_0_ is a function that counts the number of non-zeros in a *K*-element vector *u*. A convex relaxation of this constraint, adopted for the classifier used in this paper, is the mixed *ℓ*_1_/*ℓ*_2_ norm constraint [7, 23]:

(2) $$  R(\mathbf{W})=\|\mathbf{W}\|_{1,2}=\sum_{j=1}^{p} \|\mathbf{w}_{(j)}\|_{2} \leq q,  $$

where $\|\bf u\|_{2}^{2}= \sum _{k=1}^{K} {u_{k}^{2}} $ denotes the *ℓ*_2_ or Euclidean norm of *u*.

To specify the unified multiclass classifier it therefore suffices to select the sparse weights **W** and offsets *b* defining (1). These are learned from the data by solving the sparsity penalized empirical risk minimization problem:

(3) $$  \mathop {\min }\limits_{\textbf{W, \bf b}} \frac{1}{n}\sum\limits_{i = 1}^{n} V({\mathbf W}, {\mathbf b},{\mathbf x}_{i},y_{i})+\lambda R(\textbf{W}),  $$

where *R*(**W**)=∥**W**∥_1,2_ is the relaxed group sparsity inducing regularization function (2), *λ*>0 is a regularization parameter, and *V*(**W**,**b**,**x**_*i*_,*y*_*i*_) is an empirical loss function, depending on the parameters and the training data $\{{\mathbf x}_{i}, y_{i}\}_{i=1}^{n}$, that penalizes errors between the classifier output $\hat {y}_{i}$ and the true class label *y*_*i*_.

**Fig. 3:**
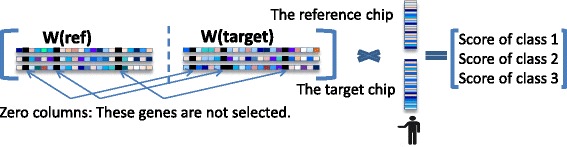
Multi-block group sparsity structure for proposed reference-based predictor. A multi-block multi-class classifier of *K*=3 classes applies a *K*×2*p* matrix **W**=[**W**(*r* *e* *f*),**W**(*t* *a* *r* *g* *e* *t*)] to the combined vector of probes on reference and target gene chips in order to form the vector of scores for each of the *K*=3 states. The two blocks of the classifier correspond to the block of weights **W**(*r* *e* *f*), applied to the reference sample, and the block of weights **W**(*t* *a* *r* *g* *e* *t*), applied to the target sample. The classifier decision rule is to assign the state label that corresonds to the maximum score. The blocks share sparsity, denoted by the black columns in the weight matrix, which designate columns that are identically zero. Variables associated with these zero columns are not selected by the classifier

As contrasted to the standard multi-class classifier, developed above, the reference-aided multi-class classifier uses a higher dimensional subject-specific input *x*_*s*_, which is a 2*p*-dimensional vector, constructed by concatenating the paired reference and target samples of subject *s*, denoted $\bf x_{s}^{ref})$ and $\bf x_{s}^{target}$, into a single vector. Note that the *j*-th and (*j*+*p*)-th elements of the vector *x*_*s*_ correspond to the same biomarker (gene probe). With the subject-specific input *x*_*s*_ the weight matrix **W** of the multi-class classifier is *K*×2*p* dimensional. Figure 3 illustrates the group sparsity constraint that enforces that **W** select only a few common biomarker variables from each pair. Such sparsity structure can be induced into the predictor by modifying the penalty function in (3) from the mixed *ℓ*_1_/*ℓ*_2_ norm (2) to the following convex function:

(4) $$  \begin{array}{c} R(\mathbf{W}) = \sum\limits_{j = 1}^{\text{p}}{\left| {\left| {\tilde{\mathbf{w}}_{(j)}} \right|} \right|_{2}} \\ \tilde{\mathbf{w}}_{(j)} = \left[\mathbf{w}_{(j)}^{T},\mathbf{w}_{(j + p)}^{T} \right]^{T}. \\ \end{array}  $$

Both the standard and the reference-aided multi-class classifier are learned by minimizing a risk function of the form (3). For the purposes of this paper, we adopt the multi-class hinge loss function *V*(**W,b**,**{***x*_*i*_**,***y*_*i*_**}**) proposed in [19] which, along with the proposed mixed *ℓ*_1_/*ℓ*_2_ norm sparsity penalty *R*, makes (3) a convex but non-smooth optimization problem. This problem can be solved with iterative optimization methods and we use an optimization algorithm, developed in [7, 23], that is based on variable splitting [24]. The optimization algorithm used in this paper is given as Algorithm 1.

A two-stage adaptive group sparsity method was used to further reduce the danger over-fitting in training the classifier. This method is an extension of the adaptive lasso [25, 26] to the group sparse multi-class classification framework developed above. The method is implemented as follows. Suppose after solving (3) we have an initial estimate **W**_*init*_,*b* of the classifier parameters. Then we refine this estimate by solving (3) once more, except in place of *R*(**W**) in (2) we use

$$R_{adapt} (\mathbf{W},\mathbf{W}_{init}) = \sum\limits_{j = 1}^{p} {\frac{\| \textbf{w}_{(j)} \|_{2} }{\| \textbf{w}_{init,(j)} \|_{2} }}, $$

for the standard classifier. A two-stage adaptive reference-aided classifier is defined similarly except that in the summand defining *R*_*adapt*_ the weights **w**_(*j*)_ and **w**_*init*,(*j*)_ are respectively replaced by $\tilde {\mathbf {w}}_{(j)}$ and $\tilde {\mathbf {w}}_{init,(j)}$.



## Results

Here we demonstrate that the reference-based predictor described in the Methods section results in improved state classification accuracy with a smaller panel of biomarkers for the challenge study dataset studied. The standard and reference-aided predictors were trained and tested separately on data from each virus category. These data are denoted H3N2, H1N1, HRV and RSV, respectively, for the pooled data from the two H3N2 studies, the pooled data from the two H1N1 studies, the pooled data from the two HRV studies, and the data from the single RSV study (see Table 1). These four virus-specific datasets consisted of *m*=29,24,31,17 subjects, respectively. Each of the virus-specific datasets was divided into *m* training-test partitions containing *m*−1 subjects for training by successively removing subjects one at a time for testing (leave-one-out partitions). For each of these subsets the predictors were trained by minimization of the empirical risk (3), using 2-fold cross-validation to first select the regularization parameter *λ* with the mixed norm sparsity constraint *R*, and an additional 2-fold cross-validation to select the regularization parameter with the adaptive sparsity inducing regularizers *R*_*adapt*_ discussed at the end of the Methods section on Prediction algorithms. The prediction performance and variable selection frequencies were assessed by averaging the predictor’s state misclassification errors over the *m* training-test partitions.

Furthermore, each of the variables in each training set was standardized to z-scores by subtracting the sample mean and dividing by the sample standard deviation, where these sample statistics were computed over the samples in the training set. The biomarkers of each subject in the each test set were standardized using the sample mean and standard deviation computed from the associated training set. To reduce possible bias due to imbalance in the numbers of samples across classes (states), at each training iteration we applied uneven cost to each sample such that the average sampling proportions among the classes were identical.

The accuracy of the reference-aided predictor is presented in row 1 of Table 2 for each of the four virus-specific datasets. For comparison the accuracy of three other predictors is shown in the remaining rows of Table 2. The proposed reference-aided predictor achieves better performance in terms of average error rates. This improvement is achieved using biomarker panels with significantly fewer genes, as compared to the standard predictor in row 2 of Table 2. The number of genes selected by the standard predictor was optimized by the two-stage adaptive cross-validation procedure described in Section Methods. Row 3 shows the performance of a constrained standard predictor when the regularization parameter is selected so that it uses approximately the same number of genes as the proposed reference-aided predictor. The actual genes selected by this constrained predictor differ from those slected by the reference-aided predictor and the performance is significantly worse than the unconstrained predictor in row 2. Row 4 shows the performance of a differential predictor that is implemented by applying the standard predictor to the difference between target and reference sample $\mathbf {x}_{s}^{\mathbf {target}}-\mathbf {x}_{s}^{\mathbf {ref}}$. This differential predictor is implemented by restricting the reference-aided predictor to the case where the reference and target weights have identical magnitudes but opposite signs. The performance in row 4 of Table is better than that of the standard predictors (rows 2 and 3) but worse than that of the proposed predictor (row 1). This indicates that simple differencing of the target and reference samples, corresponding to using all probes as contrast genes and none as reinforcement genes, leads to a less effective predictor than the one proposed in row 1 of Table 2.

**Table 2:** Average accuracy (error rate) and average size of the gene panel (number of selected genes) selected by automated predictors of infected state (class) for different viral challenges (data from DEE2/DEE5, DEE3/DEE4 and HRV-UVA/HRV-Duke were pooled and designated as H3N2, H1N1, and HRV in table). Shown are the reference-aided predictor (w/ baseline reference), the standard predictor (w/o baseline reference), the standard predictor with constraints to have the similar complexity in terms of the number of genes as the reference-aided predictor (constrained standard predictor), and the differential predictor. Across all viruses the inclusion of the baseline reference results in a decrease in error rate and a reduction in the number of genes used by the predictor. The reported accuracy and size of panel were computed by cross-validation of the predictors using leave-one-subject-out resampling to partition the data into training and target samples

Virus	H3N2	H1N1	HRV	RSV
Classes	2345	2345	2345	234
w/ baseline reference				
Error rate	0.386	0.480	0.483	0.635
Number of selected genes	200.34	287.54	287.55	238.53
w/o baseline reference				
Error rate	0.448	0.508	0.526	0.728
Number of selected genes	327.90	420.25	358.48	593.82
Constrained standard predictor				
Error rate	0.458	0.517	0.544	0.737
Number of selected genes	207.38	271.83	298.00	243.29
Differential predictor				
Error rate	0.415	0.492	0.571	0.678
Number of selected genes	481.48	1209.46	464.39	503.29

**Fig. 4:**
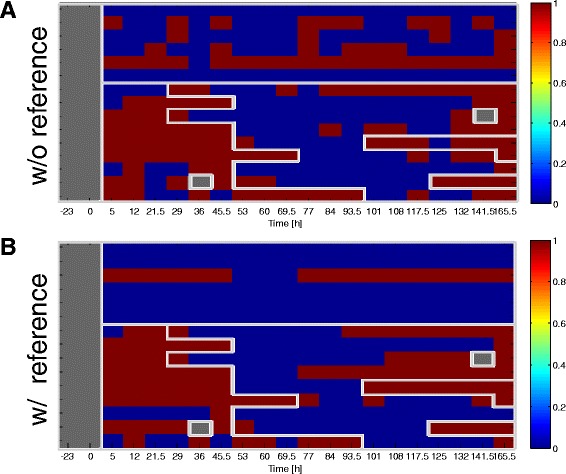
Heatmaps of predictor error rates for H3N2 DEE2 dataset. The heatmaps of the sample-specific predictor error rate for classifying states 2,3,4 and 5, defined in Fig. 2, for samples in the H3N2 DEE2 dataset. The top figure (**a**) shows the error rates of the standard predictor (no reference). The bottom figure (**b**) show the corresponding results of the reference-aided predictor

**Fig. 5:**
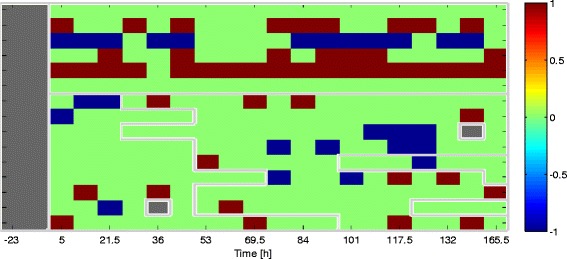
Heatmaps of predictor error rates for H3N2 DEE2 dataset. The heatmaps of the sample-specific predictor error rate for classifying states 2,3,4 and 5, defined in Fig. 2, for samples in the H3N2 DEE2 dataset. The figure shows the difference between the error rates of the standard predictor (no reference) and the error rates of the reference-aided predictor (with reference) for H3N2 DEE2. The entries in red have higher error rate using the standard predictor than the reference-aided predictor, and vice versa

The per-chip misclassification error rates, computed by aggregating over the *m* training-test partitions, are shown in Fig. 4 as heatmaps over times and subjects for each virus-specific dataset. Figures 4 and 5 show that the proposed reference-aided predictor achieves the most improvement in predicting states 2 (UnInf) and 3 (pre-acute Inf), which are the most difficult states to classify.

**Fig. 6:**
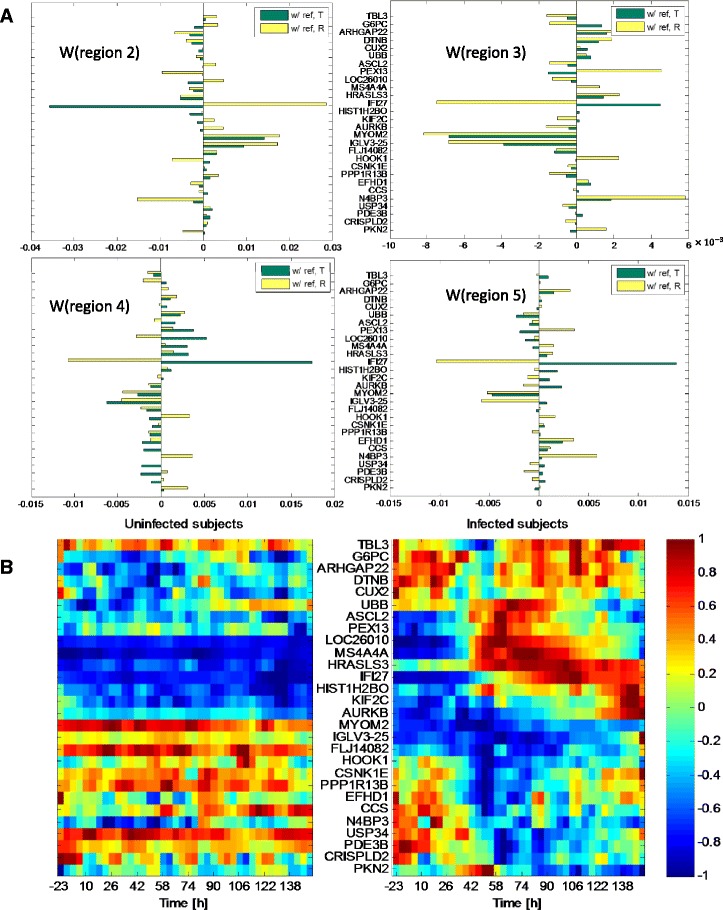
Biomarkers selected by reference-aided predictor. The top figures in (**a**) show the genes selected by the proposed reference-aided predictor with selection frequency ≥70 *%* for the 4 different score functions for states 2,3,4,5. The value of the classifier weights for each of the score functions are shown as yellow bars (weights applied to reference sample R) and green bars (weights applied to target sample T). Note that genes having yellow and green bars of opposite sign are contrasting information in R vs T while genes having these bars with the same sign are reinforcing information in R and T. The bottom figures in (**b**) show the expression of the genes shown in (**a**) averaged over the uninfected subjects (*left*) and infected subjects (*right*). The expression levels are normalized such that the maximum and minimum of each gene achieve 1 and −1 respectively. Let the averaged expression at time *t* be *z*(*t*), the maximum of *z*(*t*) be *z*_*max*_, and the minimum be *z*_*max*_. The normalized expression levels are computed as $\tilde z(t)= 2\times \left [z(t)-0.5\times (z_{\textit {max}}+z_{\textit {min}})\right ]/(z_{\textit {max}}-z_{\textit {min}})$

The different roles of the biomarkers that were automatically selected by the reference-aided predictor yields insight into these accuracy improvements. For concreteness, we focus on the H3N2 dataset. See Additional file 1 for analysis of the biomarkers selected in the HRV, RSV and H1N1 datasets. The variables that were frequently selected by the reference-aided predictors are shown in Fig. 6a for H3N2. The genes in this figure were selected with frequency at least 70 %; i.e., they were included in the trained reference-aided classifier in at least 70 *%* of the training-test partition sets. The genes are ordered according to the cluster order illustrated in panel B. The bars in Fig. 6a represent the average value of the weight *w*(*ref*) applied to a specific gene in the reference sample (R), denoted as yellow bar, and the weight *w*(*target*) applied to the same gene in the target sample (T), denoted as a green bar. These selected biomarkers can be grouped into two categories: (1) contrasting genes (R and T weights have opposite sign); and (2) reinforcing genes (R and T weights have the same sign). Contrasting genes are selected by the predictor for their differential expression between R and T, while reinforcing genes do not differentially express but rather serve to normalize the other variables (recall that the gene probes were log transformed in the RMA normalization).

An example of a contrasting gene is the interferon induced gene IFI27 which differentially expresses between R and T for states 2, 3, 4 and 5. Interestingly, the signs of the R and T weights for IFI27 are reversed in the score function for state 2 (UnInf) as compared to their signs for the score functions of the other three states (pre-acute Inf, acute-Inf, post-acute Inf): a relative decrease in IFI27 from reference to target sample induces a high UnInf score while a relative increase induces a high Inf score. An example of a reinforcing gene is the immunoglobin lambda variable IGLV3-25 that plays the role of reinforcing the UnInf state (positive contribution to state 2 score) to the detriment of the Inf states (negative contributions to state 3, 4, 5 scores). Another example, that reinforces the Inf states instead of the UnInf state, is the NEDD4 binding protein gene N4BP3. Note that some genes, e.g., PEX13 and LOC26010, take on a contrast role for some states while they take on a normalizing role for other states. Many of the genes that were selected by the reference-aided predictor were not selected by other predictors studied in Table 2. (See Sec. 4.1 in Additional file 1). Since the differential predictor can only form contrasts between reference and target gene probes none of the reinforcing genes. Indeed, we did not find the reinforcing genes, e.g., IGVL3-25, NBP3 and MYOM2 were selected by the differential predictor. The lack of reinforcement genes deprives the differential classifier of potential normalizing variables leading to poorer performance.

The average expression levels over time of the frequently selected H3N2 genes shown in Fig. 6a are shown as heatmaps in Fig. 6b, where the expression levels are averaged over the uninfected and infected subjects, respectively, in the left and right heatmaps. Notice that the reinforcing genes, such as IGVL3-25, NBP3 and MYOM2, appear to be related to susceptibility since the expression levels are substantially higher or lower in the uninfected population than in the infected population, even before viral inoculation. The expression levels of contrast genes such as IFI27, PEX13 and LOC26010, are relatively stable in the uninfected subjects but rapidly increase as an infected subject enters the acute Inf phase (roughly 40 h after inoculation).

**Fig. 7:**
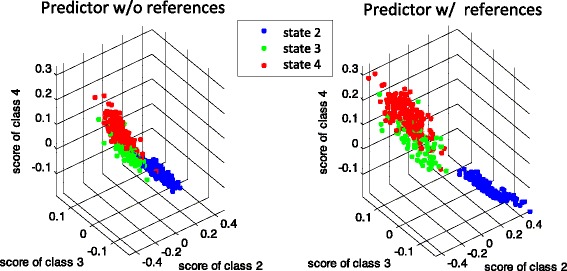
Scatter plots of the vector of scores over the H3N2 samples. Shown are scatterplots of the score functions in the multi-class classifier evaluated for the standard predictor (w/o reference) at left and for the reference-aided predictor (w reference) at right. Points with a particular denote the vector of scores for states 2, 3, and 4 for samples labeled with a particular state. The scores are better separated when the references are taken into account. Especially note that the class 2 (UnInf) scores (labeled in blue) are much better separated

The improved accuracy of the reference-aided predictor can be visualized by rendering a scatter plot of the vector of confidence scores, defined in (1), over all of the samples. In Fig. 7 the scatterplot of the *K*=3 dimensional vector of scores is shown for the H3N2 pooled challenge studies. In the scatterplot each of these vectors has been given a different color depending on the state of the particular target sample at which the score is evaluated. The right panel of Fig. 7 shows the scatterplot of confidence scores computed with the classifier weight matrix **W** by reference-aided predictor, averaged over the *m* training-test partitions. The left panel shows the associated scatterplot for the standard predictor, implemented with the average weights. Notice that the scores are better separated when the references are taken into account. Note that both with and without reference-aided training, among all pairs of states, discrimination between state 3 (pre-infection) and state 4 (acute infection) is the most difficult. This is explained by the fact that states 3 and 4 apply to the same group of subjects, namely, those that develop acute symptomatic infection with significant levels of viral shedding. For these subjects the ARI signature is developing during state 3 and comes into full bloom during state 4, thus making these states more similar to each other.

**Fig. 8:**
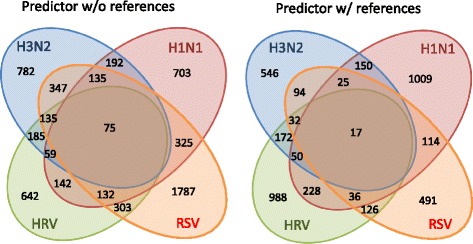
Venn diagram of the genes selected in HRV, RSV, H3N2 and H1N1 datasets. Indicated are the intersections of the genes that were selected at least once by the standard predictor (left) and reference-aided predictor (right) in each of the virus-specific datasets. The list of the genes in the intersection among all 4 datasets is listed in Table 3. These are called pan-viral predictive genes

**Fig. 9:**
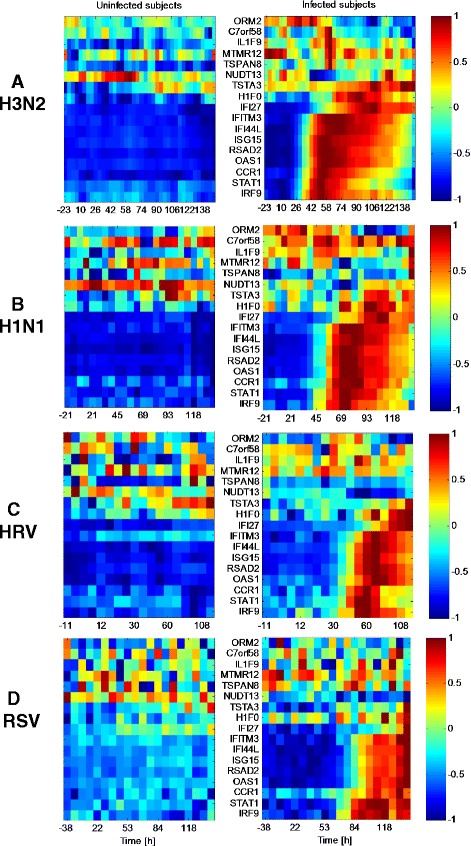
Expression profiles of reference-aided pan-viral predictive genes. Average expression profiles of the pan-viral predictive genes discovered by the reference-aided predictor (genes listed at bottom of Table 3) averaged over the uninfected subjects (*left*) and infected subjects (*right*) in each virus-specific dataset (**a** H3N2, **b** H1N1, **c** HRV, and **d** RSV). The expression levels are normalized such that the maximum and minimum of each gene achieve 1 and −1 respectively

There are common genes in the four sets of biomarkers selected by the predictors trained separately on the HRV, RSV, H3N2, and H1N1 viral-specific datasets. We call these common biomarkers pan-viral predictive genes. A Venn diagram of all the biomarkers that have been found in each virus study is shown in Fig. 8, and the pan-viral predictive genes in the intersection among all of the studies are listed in Table 3. Heatmaps, analogous to those shown in the H3N2 heatmap Fig. 6b, are shown for these pan-viral predictive genes in Fig. 9 for all 4 viral-specific datasets. Similarly to the H3N2 heatmap, some genes seem to have a reinforcement role, such as C7orf58, and others have a contrasting role, such as IFI27.

**Table 3:** Biomarkers that have been selected in every virus study. Notice that these genes are in the intersection among the 4 virus studies in Fig. 8

Method	Genes
w/o baseline reference	ACP2 ADM AFFX-r2-Bs-dap-3_at AMFR ASGR2 ATP9A BAIAP3 BTN2A2 C4BPA CDC20 CHI3L1 CYP26B1 DAP DCXR DSP EIF2AK2 ERC1 FBXL8 FOLR3 GDF9 H1F0 HMBOX1 HPCAL4 HRASLS3 IFI27 IFI44 IFI44L IFIT1 IFIT3 IGFBP6 IGFBP7 IGHV1-69 IGLV4-60 IL1F9 IRF5 IRF9 ISG15 LOC643224 LY6E MAP7 MFAP3 MICB MMP1 MX1 MYOM1 NARFL NGFRAP1 NKX3-1 NQO2 NUDT4 OAS1 OLFM1 PAPSS2 PGGT1B PODXL PRRG4 PRSS21 RGS20 RIMBP2 RSAD2 SCO2 SERGEF SERPINE2 SLC30A4 SP100 SPATA20 STAT1 TCL1B TMEM140 TNNT1 TSPAN15 TTLL4 TUBB6 UAP1L1 ZNF701
w/ baseline reference	C7orf58 CCR1 H1F0 IFI27 IFI44L IFITM3 IL1F9 IRF9 ISG15 MTMR12 NUDT13 OAS1 ORM2 RSAD2 STAT1 TSPAN8 TSTA3

## Discussion

The proposed reference-aided predictor significantly outperformed the standard predictor that does not use the reference, implemented with a single block multi-class classification algorithm. Specifically, the reference-aided predictor achieved an average (cross-validated) state prediction accuracy improvement of: 14 % for RSV, 13 % for H3N2, 9 % for HRV, and 6 % for H1N1. Remarkably, for all of these viral challenges this gain in accuracy was achieved with a smaller panel of genes: 60 % fewer for RSV, 39 % fewer form H3N2, 20 % fewer for HRV, and 31 % fewer for H1N1, as shown in Table 3. This suggests that by including such a reference sample with the target sample, the reference-aided predictor can build a more parsimonious description of the infected vs uninfected phenotypes. As seen in the previous section, this better description translates into improved prediction of infection state. There are several possible reasons that the reference-aided predictor achieves improvement in accuracy while using significantly fewer predictor variables. First, by pairing an individual’s target sample to his baseline reference sample, the reference-aided predictor turns the standard predictor into an individualized predictor. Second, the availability of a baseline sample allows the predictor to learn genes that display a contrast between an individual’s healthy baseline and sick target samples. Third, even though the reference-aided predictor has to learn twice as many coefficients, our predictor sparsity penalty forces most of these to zero, resulting in a more parsimonious predictor that minimizes overfitting errors.

Moreover, many of the genes in the panels selected by the automated reference-aided predictor and the standard predictor were different. The genes in the standard predictor were similar to the acute respiratory infection (ARI) signature reported in [4]. The reference-aided predictor selected a panel of genes that fall into two classes: 1) contrast genes that exploit the fact the the baseline reference differs significantly from the target sample; 2) reinforcement genes that do not differ significantly but are used by the classifier for baseline normalization. Specifically, for a contrast gene, the predictor forms a weighted average of the baseline and target expression levels using two coefficients having opposite sign. For a reinforcement gene these two coefficients have the same sign. We caution that these definitions only make sense when the two coefficients have similar magnitudes.

The reference-aided predictor identified the pan-viral predictive genes as some of the best subject-specific genes that either reinforce or contrast expression in the subject’s reference and target samples. Many of these genes are not included in the standard predictor that does not use a reference. Table 3) indicates that the reference-aided predictor found 8 pan-viral predictive genes (C7orf58, CCR1, IFITM3, MTMR12, NUDT13, ORM2, TSPAN8, and TSTAT3) that were not found by the standard predictor. While some of these are orthologs to genes in the standard predictor, others might represent additional pathways that can only be picked up by analysis of paired samples. For example, some studies suggest that IFITM3 is important for intrinsic viral resistance. Specifically, in vitro studies show that many pathogenic viruses’ replication can be restricted by genes in the interferon inducible transmembrane (IFITM) protein family, and it has been found that IFITM3 plays an important role in the host’s defense against influenza A virus [30]. Furthermore, it has been reported that during RSV infection deletion of CCR1 leads to attenuated pathophysiologic responses [31] and, as reported by the NCBI gene database, an important acute phase plasma protein is encoded by orosomucoid 2 (ORM2), which can be stimulated during acute inflammation and be may an important factor in immunosuppression. Other genes among the 8 genes specific to the reference-aided predictor have no obvious function in immune response but appear to have been selected to serve as normalization genes.

Many of the genes that were selected by both the predictors (with and without baseline reference) are well known transcription factors in host immune response. Specifically, interferon-stimulated genes, such as IFI44L, produce cellular factors that protect cells against invading viral pathogens [32]. OAS1 has been identified to be relevant to apoptosis, which eliminates the cells that have damaged DNA or have experienced uncontrolled proliferation [33]. Therefore, it may prevent viral replication by eliminating virus-infected cells. Indeed we observe the steady up-regulation of OAS1 during acute-infection. The role of ISG15 in innate immunity to viral infection has been studied in [34], and has been found to be highly expressed upon viral infection. IL1F9 is reported in [35] to be be up-regulated in cells involved in immune responses induced by HRV. IRF9 is one of the transcriptional activators, along with STAT1 in the ISGF3 transcriptional complex, which stimulates the expression of the interferon-inducible genes, e.g., IRF7 for antiviral responses [36]. IRF7 is one of the interferon regulatory factors, which regulates transcriptional activities to induce cellular response to the invasion of viruses. It has been reported to induce the interferon inducible genes like IFI27 in infected cells [37, 38]. Further studies suggest that IRF7 controls both the innate immunity and adaptive immunity [39, 40]. Several of the pan-viral predictive genes, e.g, IFI44L, IFI27, and OAS1 are related to type-I interferon antiviral response, and have been reported to constitute pathways regulating inflammatory response [5, 41–43].

In this paper, several viral challenge study datasets were used to demonstrate the intrinsic value of the proposed reference-aided method for biomarker selection and improved performance in predicting symptomatic infection. These findings are, of course, specific to the setting of our challenge studies and the value for clinical applications needs to be further explored. Two issues stand in the way of direct generalization of our findings to clinical medicine.

The first issue is that each enrolled subject’s healthy reference sample was collected within 24 h of exposure to the viral pathogen. An open question is whether the demonstrated performance advantages of the proposed method would generalize to the clinically relevant case where the reference sample collected in the more distant past. Such a generalization will require testing our method on observational data collected over a longer baseline period than 24 h prior to exposure. Given the expanding interest in discovery of temporal pathways for processes such as biochronicity, immunity, and aging, we can anticipate that such data will become available in the not so distant future.

The second issue is that the findings reported here are restricted to the subset of enrolled individuals who unambiguously reported health or illness, as measured by concordance between viral shedding and self-reported symptoms. All predictors have low average accuracy when ambiguous reports are included in the training data, even though the proposed reference-aided predictor maintains significant performance advantages over the other predictors to classify the state of infection (see Additional file 1: Sec. 5 for details). This poor performance on ambiguous subjects may signal the need for more complex non-linear modeling of gene expression for these subjects. On the other hand, such ambiguities may simply reflect the inadequacy of viral shedding and self reported symptom as reliable proxies for symptomatic illness.

In spite of these caveats, the framework presented here may be relevant to personalized medicine, where preventative and diagnostic medical testing could possibly benefit from availability of a recent personalized baseline reference. The reported results establish that, when used with a carefully designed classifier, inclusion of such a reference can improve the accuracy of classifiers of early onset infection based on gene expression assays. Furthermore, the variables selected by the predictor can give insight into the molecular discriminants that provide high contrast between healthy baseline and infection states. The referenced-based classifier framework we have developed can likely be extended to other diseases and diagnostic tasks, e.g., classifiying yellow fever [44, 45] or personal health monitoring [5]. For example, in a recently published paper by Chen et al. [46], the authors have demonstrated the ability of a personal ‘omics’ profile to reveal dynamic molecular and medical phenotypes by monitoring a single individual over 14 months. This might be modeled by our multi-block multi-class classifier framework, where the blocks partition the periods of health and sickness and the classes indicate different stages of infection and/or types of infection. The accuracy of such a multi-block classifier might also benefit from integration of other biomarkers, e.g., proteomic, metabolomic, antibody, into the predictor.

## Conclusions

This paper developed reference-aided prediction as way to design personalized test panels and associated predictors when one can pair a target sample with a baseline reference sample from the same subject. The framework is applicable when a population of serial samples from multiple subjects are available. The proposed referenced-aided predictor uses the framework of learning sparse linear score functions in a multi-block multi-class support vector machine (SVM). However, other types of reference-aided predictors may also be worth investigating, e.g., using multi-block non-linear kernelized multi-class classifiers or multinomial logistic classifiers.

We used a large-scale respiratory virus challenge study to illustrate the advantages of reference-aided prediction. In this predictive health problem, pre-inoculation reference (baseline) samples of each subject are incorporated into the classifier along with post-inoculation target samples. Application of the reference-aided predictor demonstrated significant improvement in the accuracy of prediction of different stages of host immune response for infected and uninfected subjects. Furthermore, it achieved this improved accuracy using fewer biomarkers than a standard predictor that does not use a reference sample. Some of the biomarkers discovered by the reference-aided predictor are genes that exhibit high contrast in expression between the target and reference samples. Other biomarkers were discovered to be low contrast genes that use the reference sample to normalize the target sample.

With minor modification, the prediction algorithm used in this paper applies to more general serially sampled diagnostic tests than the single-reference/single-target predictor. For example, consider a predictor that labels the state of a particular subject at the time a target sample is acquired using both the target sample and at least one previously acquired sample. This framework is applicable to a wide range of applications in diagnostic medicine, drug discovery and biology. For example, when using a panel of biomarkers to test for health or disease of the subject, the anterior samples might correspond to the same panel taken when the subject was at a baseline of health. When testing for the specific stage of an advancing disease the anterior samples may be panels of previously acquired target samples. It is likely that in these situations, reference-aided predictors will similarly show accuracy benefits.

## Availability of supporting data

The data has been deposited to the GEO database (accession number GSE73072).

## Additional file

Additional file 1
**Supplemental materials for the paper: An individualized predictor of health and disease using paired reference and target samples.** (PDF 14,201 kb)
