# Supplementary material for: An individualized predictor of health and disease using paired reference and target samples
Source: BMC Bioinformatics. 2016 Jan 22;17:47. doi: 10.1186/s12859-016-0889-9 (PMC4722633; doi:10.1186/s12859-016-0889-9)
Supplement: Additional file 1 — Supplemental materials for the paper: An individualized predictor of health and disease using paired reference and target samples. (PDF 14,201 kb) [file 12859_2016_889_MOESM1_ESM.pdf]

Additional file 1: Supplemental materials for the paper An individualized predictor of health and disease using paired reference and target samples

Tzu-Yu Liu[1], Thomas Burke[2], Lawrence P. Park[2], Christopher W. Woods[2],
Aimee K. Zaas[2], Geoffrey S. Ginsburg [2,*], and Alfred O. Hero[3,4,*]

[1]Electrical Engineering and Computer Science Department, University of California, Berkeley, California.
[2]Department of Medicine, Duke University, Durham, North Carolina.
[3]Electrical Engineering and Computer Science Department, University of Michigan, Ann Arbor, Michigan.
[4]Center for Computational Biology and Bioinformatics, University of Michigan, Ann Arbor, Michigan.

Contents

1 Genechip normalization **2**

2 Subject designations **2**
- 2.1 Symptom Status 2
- 2.2 Infection/Shedding Status 3

3 Five time-specific infection states **4**

4 Classification performance comparisons **5**
- 4.1 Biomarkers selected by differential predictor 5
- 4.2 Expression profiles of standard pan-viral predictive genes 9
- 4.3 Expression profiles of constrained standard pan-viral predictive genes 10
- 4.4 Expression profiles of differential pan-viral predictive genes 11

5 Prediction of ambiguous subject's state of infection and symptom **12**

*To whom correspondence should be addressed.

1 Genechip normalization

The microarray genechips are Affymetrix CEL files. We normalize the genechip and remove batch effect by the following procedures.

1. Read probe intensities from Affymetrix CEL files. We selected a custom Chip Definition File (CDF) version 10 for more accurate probe mapping to genome (Hs133Av2_Hs_ENTREZG.cdf) [1]. The task is accomplished using the function $celintensityread$ in matlab.

2. Raw gene expression profiles are preprocessed using robust multi-array (RMA) analysis [2]. We use the $affyrma$ function in matlab.

3. Possible batch effect is removed by the parametric and nonparametric empirical Bayes frameworks for adjusting data for batch effects [3].

2 Subject designations

Standard challenge study phenotypes are assigned using the criteria/algorithms described below. ID clinician will review and approve phenotype assignments generated by algorithm prior to use in analyses or manuscripts.

2.1 Symptom Status

Symptom scores are self-reported, ranging from 0 to 3, defined as follows. 0: no symptoms, 1: just noticeable, 2: bothersome but can still do activities, 3: bothersome and cannot do daily activities.

1. Eight symptoms common to all studies considered: Headache, Sore Throat, Rhinorrhea, Rhinitis, Sneezing, Cough, Myalgia, and Malaise. Exclude any additional symptom categories, e.g., fever or shortness of breath.

2. Calculate maximum symptom score per symptom per day*.

3. Sum maximum symptom scores per day*. Do not include baseline $t = 0$ symptoms (pre-inoculation or simultaneous with inoculation). Denote this as $DailyMaxSum$.

4. Sum $DailyMaxSum$ in 5-day running windows (post inoculation, do not include $t = 0$).

5. Symptomatic label is applied to participants with any 5-day symptom score sum ≥ 6.

6. Baseline adjustment: for participants with non-zero pre-inoculation symptoms ($t = 0$ or earlier) – determine using 2 methods:

   (a) No subtraction of baseline symptom scores from all time points (no baseline adjustment);

(b) Subtract baseline maximum symptom score ($t = 0$ or pre-inoculation daily max for each symptom) from all other time points, symptom by symptom (e.g., baseline Sneezing is 2, then subtract 2 from all other non-zero Sneezing symptoms from all other time points. If more than 1 pre-inoculation baseline time points available, then adjust for the max pre-inoculation symptom score for each symptom.

(c) If baseline adjusted and unadjusted symptom labels differ, flag for clinical review.

7. Symptom onset is first day of 2 or more consecutive with $DailyMaxSum$ of ≥ 2.

* For the purpose of calculating daily symptoms, "calendar days" (e.g midnight to midnight on Wed, 9/3/2014) are used rather than 24 hr periods post inoculation. For calculation of symptom onset, symptom resolution, etc, time relative to inoculation (e.g. $+12$hrs) is used.

2.2 Infection/Shedding Status

1. Virus assays performed on nasal swab samples to be considered: Virus quantitative culture (viral shedding), Virus quantitative PCR, and Virus relative quant PCR.

2. For virus quantitative culture data: Standard thresholds: for studies where we have viral culture data available (expressed in TCID50/ml or pfu/ml):

   (a) Infected if there existed greater or equal to 2 positive titer measurements that were larger than 1.25, observed at more than 24 hr post inoculation;

   (b) Infected if there existed more than 1 strong positive titer measurement that was larger than 3.0, observed at more than 24 hr post inoculation;

   (c) 2 measurable titers need not be on same or consecutive days;

   (d) Do not include Day 0 measures (0-24hrs post inoculation) since inoculum may be detected; do not include Day 28 measures where available.

3. For virus PCR data, the same thresholds as virus quantitative culture (see 2 above):

   (a) Infected if there existed more than 2 measurements that were larger than 1.25, observed at more than 24 hr post inoculation;

   (b) Infected if there existed more than 1 strong positive measurement that was greater or equal to 3.0, observed at more than 24 hr post inoculation;

   (c) PCR data should be calculated based upon standard curves, and expressed in EID50/ml or pfu/ml or pfu-e/ml;

   (d) 2 measurable titers need not be on same or consecutive days;

   (e) Do not include measures in first 24hrs post inoculation (0-24hrs) since inoculum may be detected; do not include Day 28 measures where available.

4. If both viral culture and PCR data are available, positive by one method is considered positive.

3 Five time-specific infection states

Consider the subjects whose titer scores and symptom scores agree, i.e., those who are either infected and symptomatic or uninfected and asymptomatic. We set the infection onset time and offset time for infected subjects as the time point of the first and last occurrence of measurable positive titer > 1.25 for any virus assays defined in section 2.2 respectively.

1. Samples acquired before inoculation, are labeled as baseline references (state 1);
2. Samples from the uninfected subjects after inoculation are labeled as uninfected (state 2);
3. Samples acquired before the onset time from infected subjects after inoculation are labeled as pre-acute infection (state 3);
4. Samples collected between onset and offset time points ($\geq$ onset time and $\leq$ offset time) are labeled as acute-infection (state 4);
5. Samples obtained after the offset time ($>$ offset time) are labeled as the post-acute infection (state 5).

4 Classification performance comparisons

4.1 Biomarkers selected by differential predictor

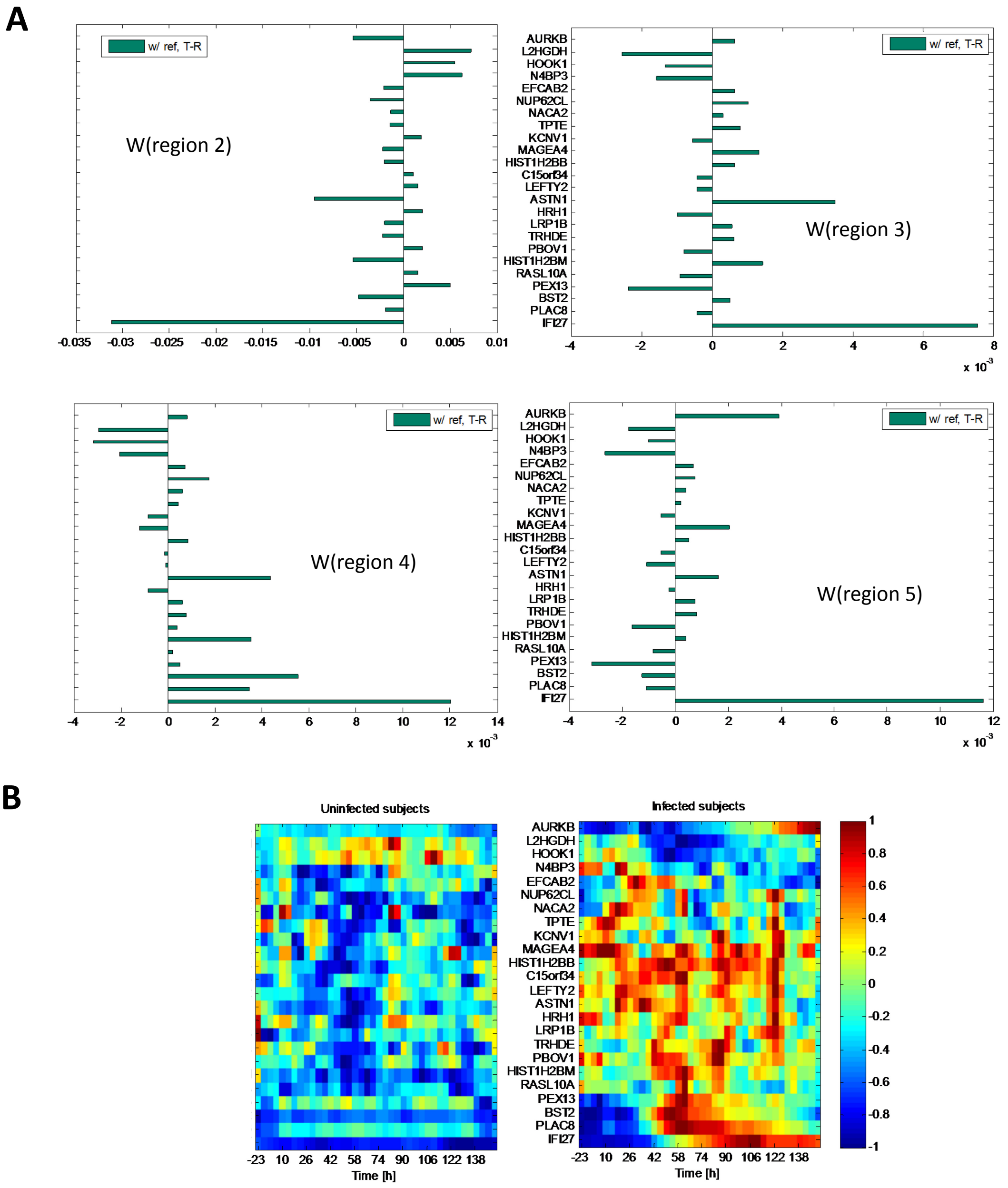

Figure 1: Biomarkers selected by differential predictor for H3N2 dataset. The top figures in (A) show the genes selected by the proposed reference-aided predictor with selection frequency $\geq 80\%$ for the 4 different score functions for states 2,3,4,5. The value of the classifier weights for each score functions are shown as green bars (weights applied to target sample T).

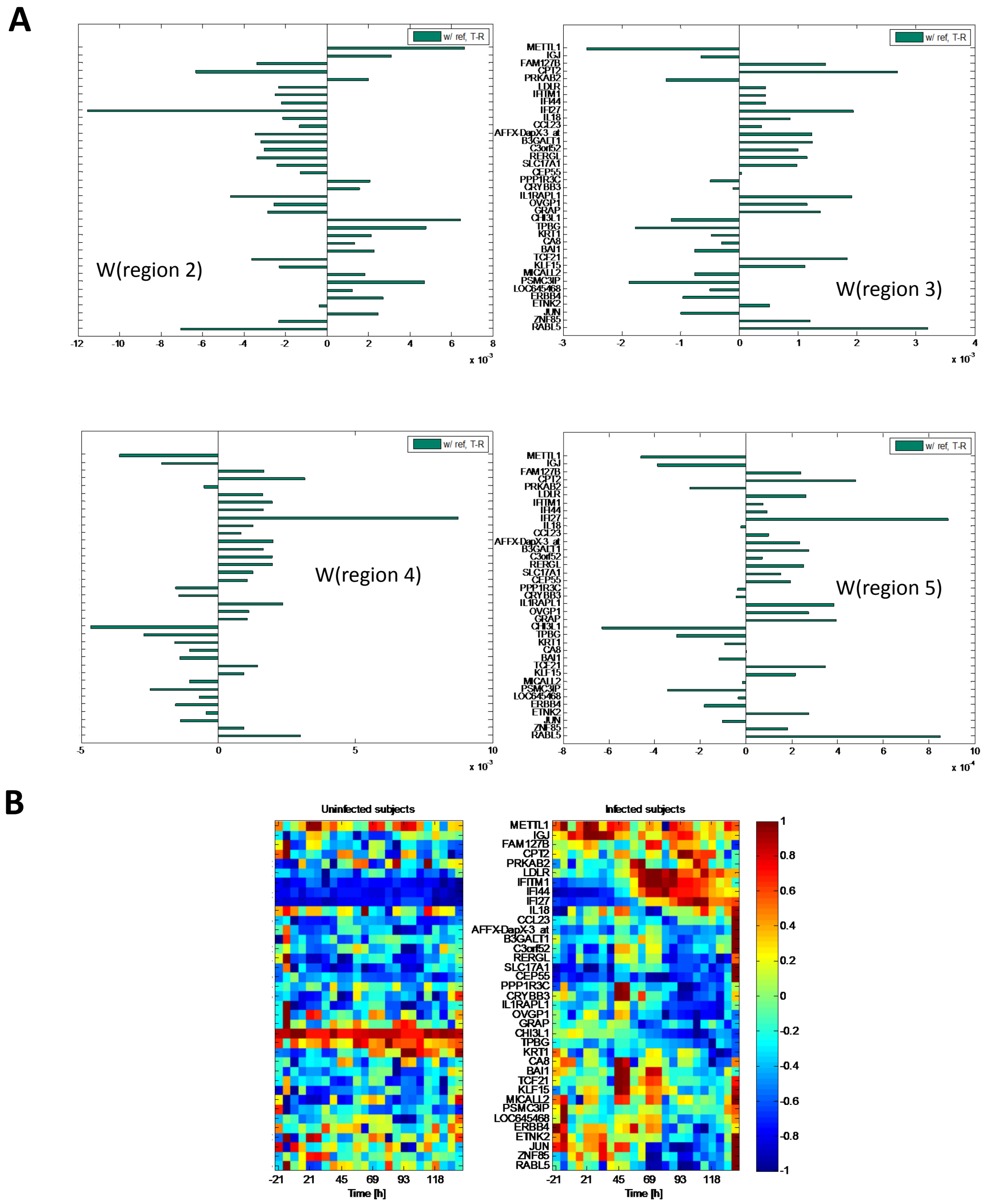

Figure 2: Biomarkers selected by differential predictor for H1N1 dataset. The top figures in (A) show the genes selected by the proposed reference-aided predictor with selection frequency $\geq 95\%$ for the 4 different score functions for states 2,3,4,5. The value of the classifier weights for each score functions are shown as green bars (weights applied to target sample T).

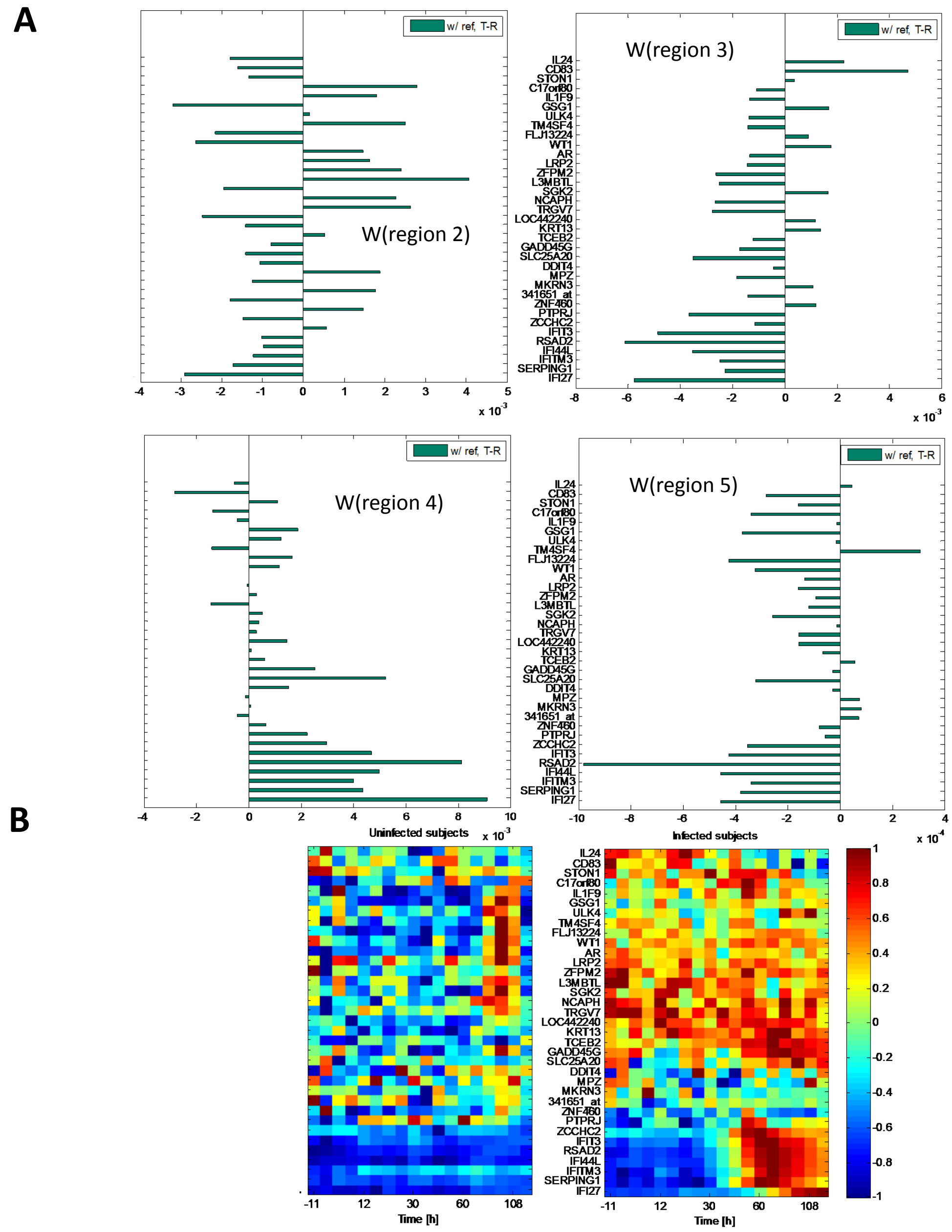

Figure 3: Biomarkers selected by differential predictor for HRV dataset. The top figures in (A) show the genes selected by the proposed reference-aided predictor with selection frequency $\geq 95\%$ for the 4 different score functions for states 2,3,4,5. The value of the classifier weights for each score functions are shown as green bars (weights applied to target sample T).

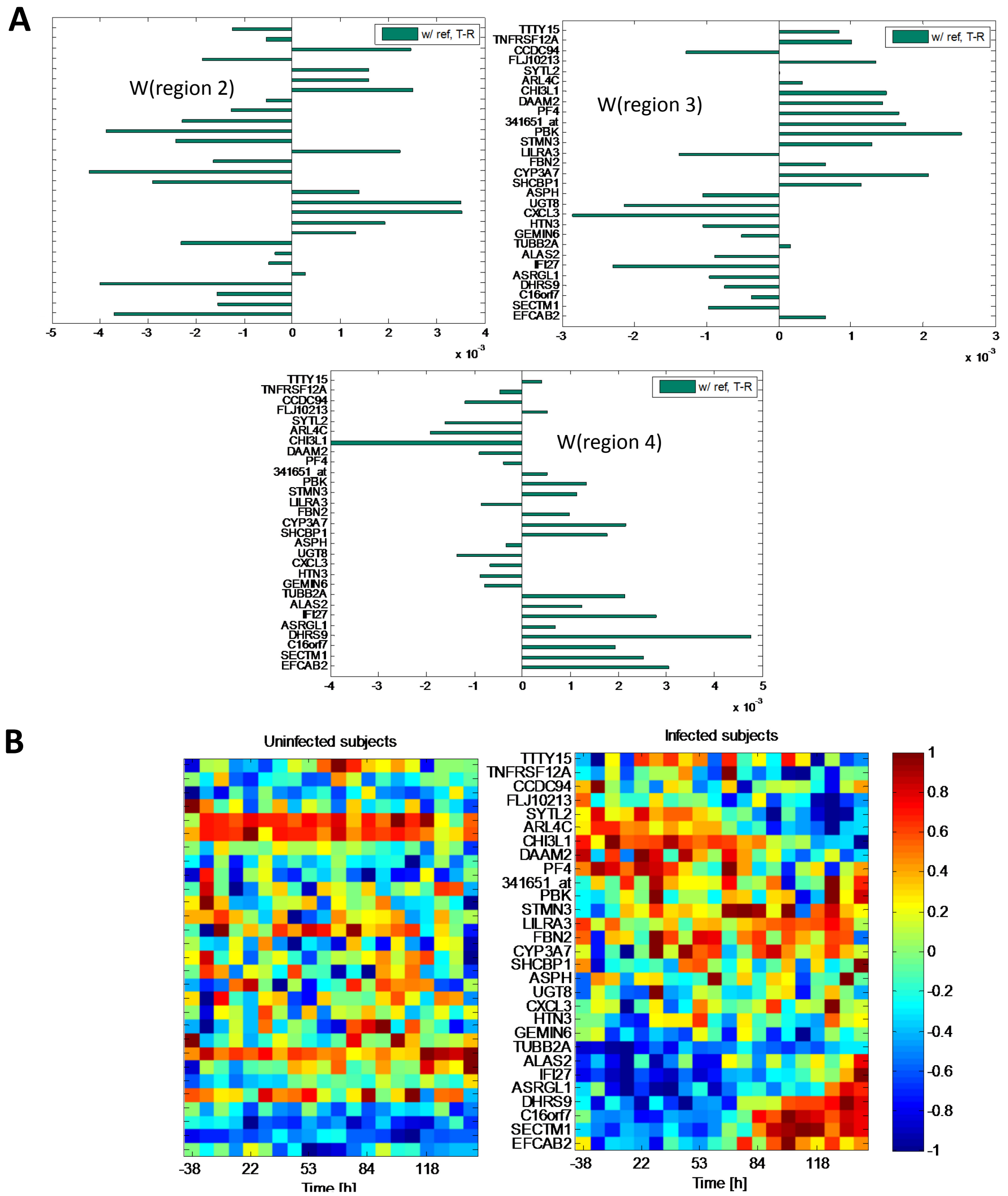

Figure 4: Biomarkers selected by differential predictor for RSV dataset. The top figures in (A) show the genes selected by the proposed reference-aided predictor with selection frequency $\geq 90\%$ for the 3 different score functions for states 2,3,4. The value of the classifier weights for each score functions are shown as green bars (weights applied to target sample T).

4.2 Expression profiles of standard pan-viral predictive genes

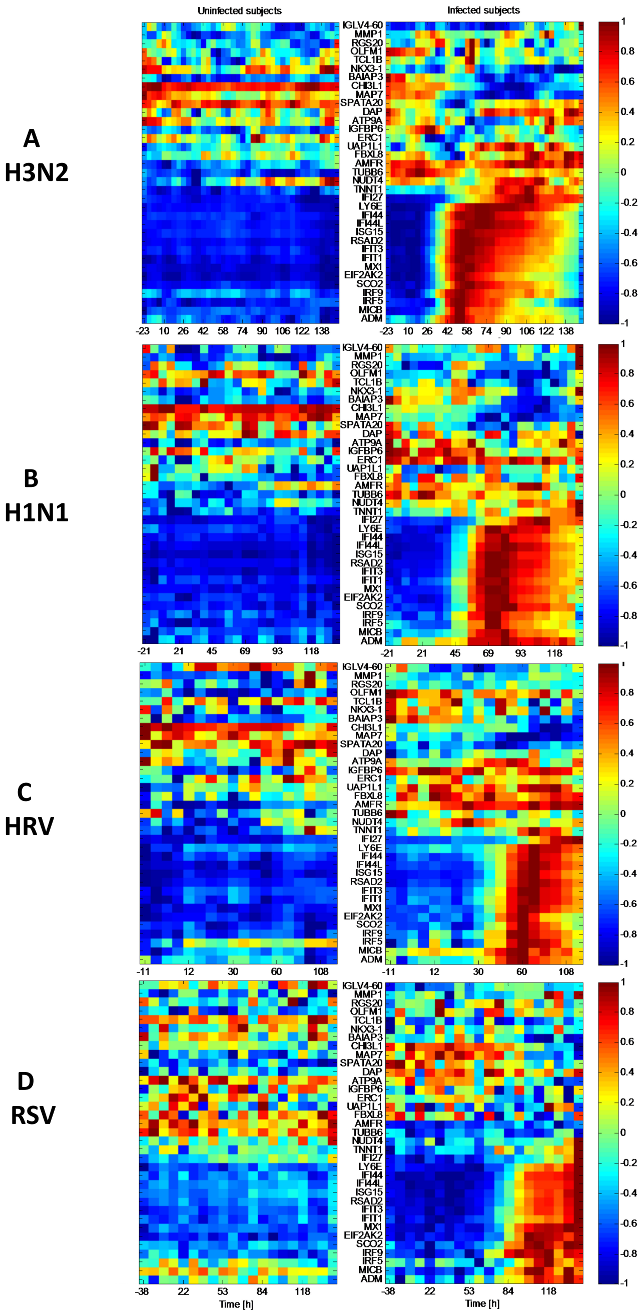

Figure 5: Expression profiles of standard pan-viral predictive genes. Average expression profiles of the top 5 % pan-viral predictive genes discovered by the standard predictor averaged over the uninfected subjects (left) and infected subjects (right) in each virus-specific dataset ((A) H3N2, (B) H1N1, (C) HRV, and (D) RSV). The expression levels are normalized such that the maximum and minimum of each gene achieve 1 and -1 respectively.

4.3 Expression profiles of constrained standard pan-viral predictive genes

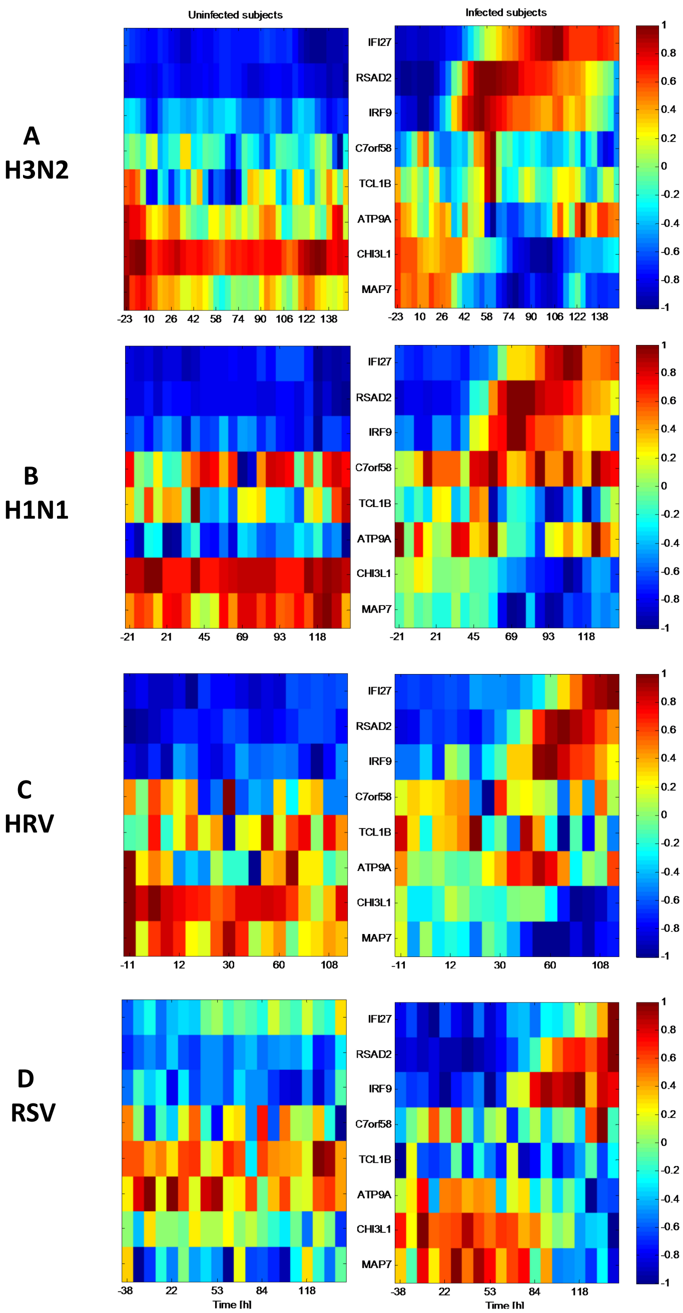

Figure 6: Expression profiles of standard pan-viral predictive genes. Average expression profiles of the top 5 % pan-viral predictive genes discovered by the standard predictor averaged over the uninfected subjects (left) and infected subjects (right) in each virus-specific dataset ((A) H3N2, (B) H1N1, (C) HRV, and (D) RSV). The expression levels are normalized such that the maximum and minimum of each gene achieve 1 and -1 respectively.

4.4 Expression profiles of differential pan-viral predictive genes

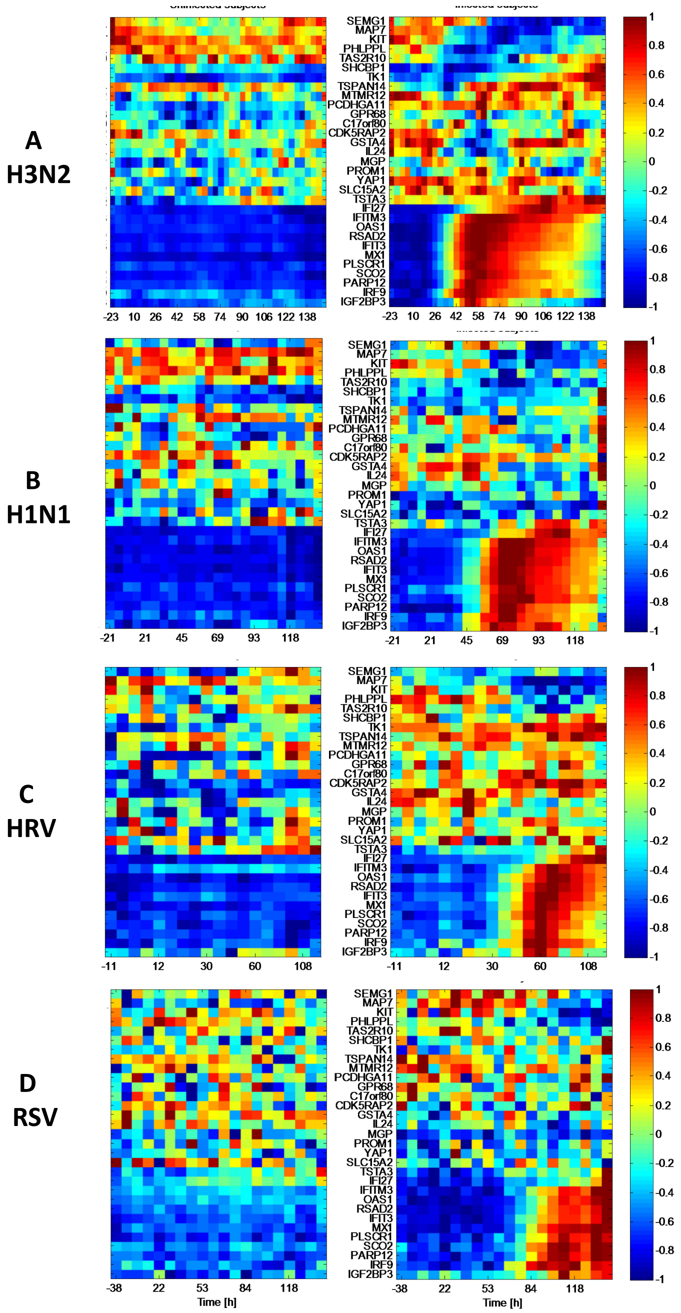

Figure 7: Expression profiles of standard pan-viral predictive genes. Average expression profiles of the top 10 % pan-viral predictive genes discovered by the standard predictor averaged over the uninfected subjects (left) and infected subjects (right) in each virus-specific dataset ((A) H3N2, (B) H1N1, (C) HRV, and (D) RSV). The expression levels are normalized such that the maximum and minimum of each gene achieve 1 and -1 respectively.

5 Prediction of ambiguous subject's state of infection and symptom

In the main text we have we excluded clinically ambiguous subjects due to inconsistencies between their declared symptomatic status and measured shedding status. Here we apply the predictors trained using the unambiguously healthy and unambiguously ill subjects to predict the infected/uninfected states of the ambiguous subjects. These are different predictors trained for each viral challenge. Not surprisingly, the states of the clinically ambiguous subjects are difficult to predict even when using the reference aided classifer. Table 1 shows that, as compared to the standard classifier, the reference aided classifier attains a lower error rate than the standard classifier for H1N1 and HRV but not for the other viral species. However, the reference-aided classifier does achieve a reduction in the average classification error. When averaged over all the different viral species (rows of Table 1), the mean prediction accuracies on the uninfected but symptomatic subjects are 0.57 by the standard predictors and 0.49 by the reference-aided predictors. The corresponding mean accuracies on the infected but asymptomatic subjects are 0.66 by the standard predictors and 0.57 by the reference-aided predictors.

Table 1: Average accuracy (error rate) for prediction of infected vs uninfected state for different viral challenges (data from DEE2/DEE5, DEE3/DEE4 and HRV-UVA/HRV-Duke were pooled and designated as H3N2, H1N1, and HRV in table). Shown are the standard predictor (w/o baseline reference), the reference-aided predictor (w/ baseline reference) trained using the unambiguously healthy and unambiguously ill subjects and applied to the ambiguous subjects. The shedding status defined in Sec. 2.2 is used to define the ground truth state of infection. The predictors classify the ambiguous subjects as either infected or uninfected subjects and shown in the table are the error rates relative to ground truth.

virus	H3N2		H1N1		HRV		RSV	
state	Uninf/Sx	Inf/Asx	Uninf/Sx	Inf/Asx	Uninf/Sx	Inf/Asx	Uninf/Sx	Inf/Asx
number of subjects	5	4	6	13	9	4	0	3
w/o baseline reference error rate	0.24	0.64	0.34	0.82	0.69	0.51		0.17
w/ baseline reference error rate	0.30	0.72	0.46	0.65	0.47	0.21		0.52

Next, we apply the predictors trained using the unambiguously healthy and unambiguously ill subjects to predict the symptomatic/asymptomatic states of the ambiguous subjects. Table 2 shows that, in opposition to Table 1, the Sx/Asx reference aided predictor reduces the error for H3N2 and RSV but not for H1N1 and HRV. This dichotomy might be partially explained by the fact that symptoms were milder in the H1N1 and HRV cohorts than in the H3N2 and RSV cohorts. Therefore, a larger number of H1N1 and HRV subjects who were clearly infected may not have accurately reported their symptoms.

Unlike for infected state prediction, shown in Table 1, the referenced based symptom predictor does not reduce the average error when averaged over all viral challenge cohorts. The overall

prediction error on all ambiguous subjects is 0.43 using the standard predictors, and 0.49 using the reference-aided predictors. The accuracies on the uninfected but symptomatic subjects are 0.53 by the standard predictors and 0.58 by the reference-aided predictors. The accuracies on the infected but asymptomatic subjects are 0.34 by the standard predictors and 0.43 by the reference-aided predictors.

Table 2: Average accuracy (error rate) for prediction of symptomatic vs asymptomatic state for different viral challenges (data from DEE2/DEE5, DEE3/DEE4 and HRV-UVA/HRV-Duke were pooled and designated as H3N2, H1N1, and HRV in table). Shown are the standard predictor (w/o baseline reference), the reference-aided predictor (w/ baseline reference) trained using the unambiguously healthy and unambiguously ill subjects and applied to the ambiguous subjects to classify the state of symptoms, i.e., the predictors classify the ambiguous subjects as either symptomatic or asymptomatic subjects. The ground truth symptom states of the subjects were determined from self-reported symptoms as described in Sec 2.1.

virus	H3N2		H1N1		HRV		RSV	
state	Uninf/Sx	Inf/Asx	Uninf/Sx	Inf/Asx	Uninf/Sx	Inf/Asx	Uninf/Sx	Inf/Asx
number of subjects	5	4	6	13	9	4	0	3
w/o baseline reference error rate	0.76	0.36	0.66	0.18	0.31	0.49		0.83
w/ baseline reference error rate	0.70	0.27	0.54	0.35	0.53	0.79		0.48

References

[1] Dai, M., Wang, P., Boyd, A.D., Kostov, G., Athey, B., Jones, E.G., Bunney, W.E., Myers, R.M., Speed, T.P., Akil, H., Watson, S.J., Meng, F.: Evolving gene/transcript definitions significantly alter the interpretation of genechip data. Nucleic acids research **33**(20), 175–175 (2005)

[2] Bolstad, B.M., Irizarry, R.A., Åstrand, M., Speed, T.P.: A comparison of normalization methods for high density oligonucleotide array data based on variance and bias. Bioinformatics **19**(2), 185–193 (2003)

[3] Johnson, W.E., Li, C., Rabinovic, A.: Adjusting batch effects in microarray expression data using empirical bayes methods. Biostatistics **8**(1), 118–127 (2007)
